# Two Decades of Huntington’s Disease in Varna, Bulgaria: A Retrospective Single-Centre Study of Clinical Trends and Challenges

**DOI:** 10.3390/neurolint17060095

**Published:** 2025-06-18

**Authors:** Mariya Levkova, Mihael Tsalta-Mladenov, Milena Stoyanova, Mari Hachmeriyan, Lyudmila Angelova, Ara Kaprelyan

**Affiliations:** 1 Department of Medical Genetics, Medical University Varna, Marin Drinov Str 55, 9000 Varna, Bulgaria; 2 Laboratory of Medical Genetics, University Multiprofile Hospital for Active Treatment “St. Marina”, Hristo Smirnenski Blv 1, 9000 Varna, Bulgaria; 3 Department of Neurology and Neuroscience, Medical University Varna, Marin Drinov Str 55, 9000 Varna, Bulgaria; 4 Second Clinic of Neurology with ICU and Stroke Unit, University Multiprofile Hospital for Active Treatment “St. Marina”, Hristo Smirnenski Blv 1, 9000 Varna, Bulgaria

**Keywords:** Huntington’s disease, epidemiology, genetic counselling, neurodegeneration

## Abstract

**Background**: Huntington’s disease (HD) is a progressive, autosomal dominant neurodegenerative disorder caused by an expanded CAG repeat in the *HTT* gene. Despite advances in understanding its molecular basis, epidemiological data in many countries, including Bulgaria, remain limited. This study aims to present clinical and genetic findings from a 20-year single-centre cohort. **Methods**: A retrospective review was conducted of patients evaluated for HD at the University Hospital “St. Marina” in Varna between 2004 and 2024. Data included demographics, CAG repeat length, clinical features, imaging, and psychiatric assessments. Statistical analysis focused on correlations between variables, with significance set at *p* < 0.05. **Results**: Out of 79 referred individuals, 43 were molecularly confirmed. The mean age of onset was 43 years, with a four-year diagnostic delay. The average CAG repeat length was 44.6, though two symptomatic patients had reduced penetrance alleles (38 and 39 repeats). Cognitive and psychiatric symptoms were each present in 72% of cases. Depression was significantly more prevalent in women (*p* = 0.011). Most patients had a positive family history, predominantly maternal. **Conclusions**: Our findings highlight diagnostic delays, gender-specific psychiatric vulnerabilities, and the importance of personalized care. Improved access to genetic counselling and early diagnosis are essential for optimizing outcomes.

## 1. Introduction

Huntington’s disease (HD), also known as Huntington’s chorea, is a progressive neurodegenerative disorder first described by George Huntington in 1872 [1]. Over a century later, the identification of the causative gene, *HTT*, sparked a surge in molecularly confirmed diagnoses [2]. HD is now well-characterized as an autosomal dominant genetic disorder caused by an expanded CAG trinucleotide repeat in exon 1 of the *HTT* gene [3]. The disorder’s primary clinical manifestations include involuntary choreiform (dance-like) movements, cognitive decline leading to dementia, and a range of behavioural and psychiatric disturbances. HD exhibits incomplete penetrance with 36–39 CAG repeats, but full penetrance at 40 or more repeats [3]. The mutation is characterized by intergenerational instability; the number of CAG repeats can change between generations, often leading to earlier onset and increased disease severity with longer repeat lengths—a phenomenon known as anticipation [3].

While CAG repeat length is a major determinant of age at onset, significant variability exists, particularly in individuals with fewer than 60 repeats. The somatic expansion of the CAG repeat within the brain, especially in the striatum and cortex, is strongly correlated with earlier disease onset. This expanded CAG repeat is somatically unstable and actively transcribed/translated, suggesting that this instability itself contributes to the age of onset [4].

Recent research proposes a stage model of CAG amplification in striatal projection neurons. An initial slow expansion phase (36–80 CAGs) is followed by a rapid expansion phase (potentially exceeding 150 CAGs), contributing to the variable age at onset. This progressive somatic expansion of long DNA repeats is a key driver of neurodegeneration in Huntington’s disease [5].

Despite significant advances in understanding the molecular mechanisms of HD, epidemiological data remain limited, with existing studies covering only 13 European countries and no available data from ours [6]. Given the disease’s rarity, with a prevalence of approximately 5.7 per 100,000 people [7], multinational epidemiological data is needed. This article aims to present 20 years of Huntington’s disease patient data from our single tertiary centre and analyze the observed trends within our community.

## 2. Materials and Methods

This retrospective, single-centre study was conducted to analyze the epidemiological, clinical, genetic, and neuroimaging characteristics of patients with Huntington’s disease evaluated at the University Hospital “St. Marina”, Varna, Bulgaria. The study population included all individuals with a confirmed molecular diagnosis of HD between 1 January 2004, and 31 December 2024. Data were extracted from electronic medical records and genetic testing reports.

Clinical Data Collection: All patients were evaluated by an experienced multidisciplinary team, including a neurologist, medical genetic counsellor, and psychiatrist. Comprehensive clinical assessments were performed, encompassing the following:

Neurological Assessment: This included the Unified Huntington’s Disease Rating Scale (UHDRS), specifically the Total Motor Score (TMS), Total Functional Capacity (TFC), the Independence Scale, and the Diagnostic Confidence Level (DCL), which ranges from 0 (normal) to 4 (clinically definite HD). An average DCL score of 2.88 in our sample reflects a probable diagnosis, suggesting motor abnormalities that are likely due to HD but not yet unequivocal for a definite diagnosis. Cognitive function was assessed using the Bulgarian version of the Mini-Mental State Examination (MMSE; range: 0–30), which has been validated for the local population. A score below 24 is generally considered indicative of cognitive impairment in this context.

Psychiatric Assessment: Depression was assessed using the Beck Depression Inventory, Second Edition (BDI-II), which is validated for use in the Bulgarian population. Additionally, symptoms of apathy and sleep disturbances were evaluated through clinician-administered interviews and patient self-reports. However, no standardized scales were applied for these additional symptoms.

Demographic and Clinical Variables: Collected variables included age, sex, ethnicity, age at symptom onset, age at genetic testing, family history of HD (including pedigree data), and key features of disease progression (e.g., motor symptoms, cognitive decline, and psychiatric manifestations).

Genetic Data: Genetic testing involved the determination of the number of CAG trinucleotide repeats in the HTT gene. A polymerase chain reaction (PCR)-based assay was used, followed by Sanger sequencing for confirmation and precise sizing of the repeat expansion.

Neuroimaging Data: Brain imaging studies were reviewed when available, including structural MRI and (18)F-FDG PET scans. Imaging data were assessed by board-certified neuroradiologists and nuclear medicine specialists. Caudate volume was evaluated through visual inspection and manual tracing techniques on coronal MRI slices. Quantitative volumetric or voxel-based PET analyses were not feasible due to the retrospective and clinically oriented nature of the dataset.

Statistical Analysis: Data were anonymized and analyzed using IBM SPSS Statistics, Version 26. Continuous variables were tested for normality using the Shapiro–Wilk test. Normally distributed variables were compared using independent-samples *t*-tests, while categorical variables (e.g., the presence or absence of depression) were compared using chi-square tests of independence. Effect sizes were reported as Cohen’s d for *t*-tests and Cramér’s V for chi-square tests, along with 95% confidence intervals for mean differences. A two-sided *p*-value of <0.05 was considered statistically significant. No corrections for multiple comparisons were applied, as this was an exploratory study.

## 3. Results

A total of 79 patients were referred to the genetic counselling unit based on a clinical diagnosis of Huntington’s disease. However, not all patients consented to molecular-genetic testing, primarily because it is a paid service in our country. During the study period, 43 patients tested positive for Huntington’s disease (Supplementary Table S1). All 43 participants completed the full clinical and psychiatric assessments. The majority (81%) were referred for genetic counselling by a neurologist due to clinical suspicion, while the remaining patients were self-referred or tested following evaluation by a genetic counsellor. Of the cohort, 24 patients (56%) were women and 19 (44%) were men. Regarding employment status, 21 individuals (49%) were unemployed, 13 (30%) were employed, and 9 (21%) were retired.

**Figure 1:**
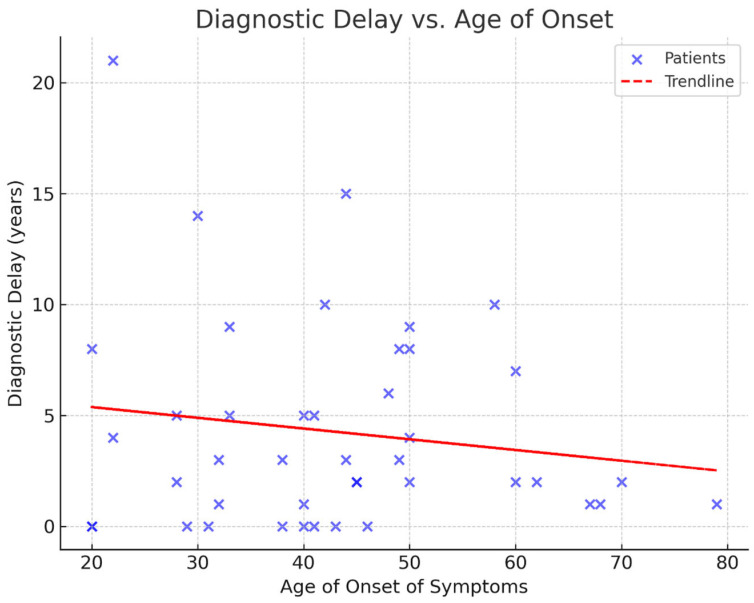
A scattered plot illustrating the diagnostic delay in years.

The mean age at genetic testing was 47 years, while the mean age of symptom onset was 43 years. The youngest patient was 20 years old, and the oldest was 80 years old. Our data indicate an average diagnostic delay of four years (Figure 1). The mean number of CAG repeats was 44.6, with the highest recorded repeat length being 55. Interestingly, two patients developed symptoms despite having repeat numbers below the typical pathogenic threshold of 40—specifically, 38 and 39 repeats. These individuals had a late age of onset at 60 and 70 years, respectively.

A positive family history of Huntington’s disease was reported in 33 patients (77%). Among those without a documented family history, two had a parent who had died before the age of 40, potentially obscuring inheritance patterns. In 23 individuals (58%), the positive family history was on the maternal side. Additionally, 13 patients (30%) had more than one affected relative.

Cognitive impairment, ranging from mild to moderate (as assessed by the Mini-Mental State Examination), was observed in 31 patients (72%). Similarly, 31 patients (72%) exhibited psychiatric symptoms, primarily depression and apathy. Sleep disturbances and/or weight loss were reported by 16 patients (37%), while the remaining 27 individuals (63%) denied such issues. Only 28 (65%) individuals reported themselves as functionally independent, despite reporting a higher number of patients with psychiatric symptoms. This difference reflects variation in self-perceived independence rather than missing data or participant dropout. The mean DCL of the Unified Huntington’s Disease Rating Scale was 2.88 (Supplementary Table S1).

**Figure 2:**
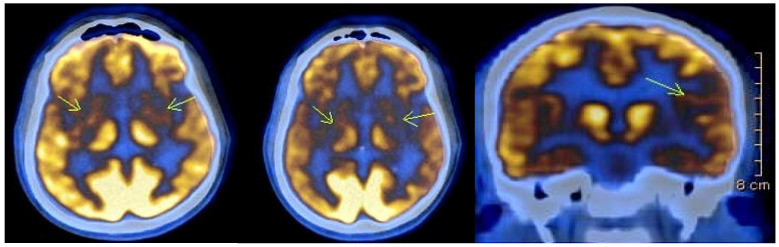
(18)F-FDG PET images of a 35-year-old patient with Huntington’s disease showing severely reduced to absent metabolic activity (indicated with arrows) bilaterally in the striatum. A follow-up scan one year later revealed additional hypometabolism (indicated with arrows) in the left temporoparietal region. Imaging was obtained as part of routine clinical care, and detailed acquisition parameters (e.g., isotope dose and time post-injection) were not available due to the retrospective nature of the data.

Imaging studies were conducted in 27 individuals, and revealed typical findings, including atrophy of the caudate nucleus and cortical atrophy in most patients. Eight of the affected individuals underwent Fluorine-18 Fluorodeoxyglucose Positron Emission Tomography (18)F-FDG PET) as part of a separate ongoing research project. In seven of them (87.5%), the scans revealed areas of significantly reduced or absent metabolic activity in the caudate nucleus and putamen bilaterally. Additionally, cortical hypometabolism was observed in 50% of cases in the left superior parietal region, in 37.5% of cases in the right superior parietal region, and in 12.5% of cases in the left superior and inferior frontal regions. One of the patients was followed up after one year, and the results showed further reduction to absence of metabolic activity bilaterally in the striatum, as well as in the left temporoparietal region (Figure 2).

Regarding pharmacological management, 23 patients (54%) were prescribed an antipsychotic drug upon discharge, while 10 (23%) received antidepressants. Another nine patients (21%) were not prescribed any medications at the time of evaluation, while the remaining individuals were treated with anxiolytics or tetrabenazine.

We assessed sex-related differences in clinical features using independent-samples *t*-tests for continuous variables and chi-square tests for categorical outcomes. For depression scores, an independent-samples *t*-test showed that women had significantly higher mean scores (M = 2.00; SD = 0.60) compared to men (M = 1.62; SD = 0.49) (t(41) = 2.78; *p* = 0.008; Cohen’s d = 0.7; 95% CI [0.10, 0.66]).

To assess categorical clinical features (coded as 1 = No, 2 = Yes), we performed chi-square tests. There was a significant association between sex and depressive symptoms (χ^2^(1, N = 43) = 4.79; *p* = 0.029; Cramér’s V = 0.33), suggesting women more frequently reported depression. No significant sex differences were found for cognitive decline (χ^2^ = 0.018; *p* = 0.892) or independence (χ^2^ = 0.00; *p* = 1.000).

No significant associations were found between sex and age of onset, diagnostic delay, or cognitive impairment severity (*p* > 0.05).

## 4. Discussion

Our study provides insights into the clinical, genetic, and treatment characteristics of patients diagnosed with HD in our single centre. The data showed that the mean DCL score was close to 3, indicating that most patients exhibited motor abnormalities highly suggestive of HD, with a 90–98% confidence level [8]. Given that motor dysfunction is classically considered the defining clinical onset of HD, it is expected that it is often the first noticeable symptom [9]. However, the nearly four-year diagnostic delay observed in our cohort is noteworthy, particularly given that most patients had a positive family history and exhibited a clinically typical presentation. While the high cost of molecular-genetic testing, especially during the early years of the study period, likely played a role, attributing the delay solely to financial barriers oversimplifies a more complex issue. Additional contributing factors may include clinical uncertainty, especially in cases with atypical onset; psychiatric misdiagnoses, which are common in the prodromal phase of Huntington’s disease; stigma associated with the diagnosis; and healthcare access inequalities, particularly in under-resourced or rural areas. While additional clinical scales for HD assessment have been introduced over time [10], our study utilized the DCL scale, as it was the standard measure when our data collection began in 2004.

Our findings revealed statistically significant sex-dependent differences in depressive symptoms, with women being more frequently affected than men. This result is consistent with prior studies and supports the need for a gender-sensitive approach to Huntington’s disease (HD) management [11]. While the precise biological and psychosocial mechanisms remain unclear, several factors may contribute to this discrepancy. From a neuroendocrine perspective, fluctuations in estrogen and other gonadal hormones have been associated with increased vulnerability to affective disorders in women [12]. In addition, sociocultural expectations often position women as primary caregivers, even when they themselves may be at risk of or affected by HD—a dynamic that amplifies both emotional and physical stress [13]. Furthermore, internalized stigma, differences in coping and help-seeking behaviour, and societal attitudes toward mental health may influence how depressive symptoms are reported, perceived, and managed [14]. There are conflicting reports suggesting that disease stage, rather than sex, is associated with depressive symptoms. However, that particular study analyzed data primarily from Western European countries. Given the substantial differences in healthcare system organization between Eastern and Western Europe, this may have influenced the findings [15].

A positive family history was identified in 77% of patients, consistent with the autosomal dominant inheritance pattern of Huntington’s disease [16]. However, two individuals with no reported family history had a parent who died before the age of 40, highlighting the challenges of incomplete family histories due to the late onset of the disorder.

Interestingly, we observed a maternal predominance in inheritance (58%). Offspring of affected mothers are generally more likely to exhibit stable repeat lengths [11,16]. However, given the relatively small sample size and the retrospective nature of the study, this finding should be interpreted with caution, as it may reflect a random variation rather than a true biological trend. Moreover, due to the unavailability of most parents for molecular-genetic testing, we were unable to confirm inheritance patterns directly. Existing research suggests that in maternal transmissions, repeat-length changes may also be influenced by the sex of the offspring, with a tendency for expansion in males and contraction in females [11,16]. Also, there are other genes which are potentially involved in the clinical presentation and age of onset, located at chromosomes four and six [17]. As more data accumulates on the molecular mechanisms of HD, genetic counselling and predicting disease severity in future generations would become increasingly complex.

The mean age of symptom onset in our cohort (43 years) is consistent with previous reports [6]. However, the observation of two symptomatic individuals with 38 and 39 CAG repeats raises important considerations regarding the penetrance of reduced-penetrance alleles [18]. While CAG expansions of ≥40 are generally considered fully penetrant, repeat lengths between 36 and 39 fall into a reduced penetrance category. Established models, such as those by Langbehn et al. [19], estimate that the probability of clinical onset by age 82 is below 90% for individuals with 39 repeats and approximately 60% for those with 38 repeats [10,19].

The presence of definitive clinical symptoms in our patients with 38 and 39 repeats suggests the potential involvement of additional genetic modifiers (e.g., MSH3 and FAN1) or environmental and epigenetic factors influencing disease onset and expressivity [20]. Recent studies support this hypothesis, highlighting variability in symptom onset even within families sharing the same CAG expansion [21]. Furthermore, individuals with reduced penetrance alleles have occasionally presented with atypical clinical features or phenotypic overlap with other neurodegenerative conditions, including ALS [22], complicating both diagnosis and counselling.

These findings reinforce the need for a nuanced interpretation of low-penetrance CAG repeats, particularly in clinical and predictive genetic counselling. They also support the growing recognition that current penetrance models may benefit from incorporating modifier effects, ancestry-related variability, and longitudinal phenotype–genotype correlation data [19,21].

Despite ongoing clinical trials, no disease-modifying treatment exists to stop or slow HD progression. Current management focuses on symptom relief and maximizing functionality for as long as possible [23]. However, the progressive nature of the disease significantly impacts patients’ independence and work status. In our study, nearly half of the patients were unemployed, and approximately 40% required assistance with daily activities, reflecting the increasing financial burden on affected families. The costs associated with HD extend beyond direct medical expenses, including medications, outpatient services, hospital visits, and caregiver support. According to published data, the estimated annual costs for HD would increase even further with the advancing of the disorder [24]. These findings underscore the urgent need for better support systems and policy interventions to alleviate the economic impact on patients and their families.

Our findings should be interpreted in light of several limitations. First, the cohort originates from a single tertiary centre in Varna and includes only patients who either self-referred or were specifically referred by neurologists familiar with Huntington’s disease. This introduces a potential selection bias toward individuals with more pronounced or classical phenotypes, likely under-representing those with milder or atypical presentations. Second, the retrospective design of the study means that key clinical variables, such as age at motor onset, psychiatric prodromes, and family history, rely on patient or caregiver recall and medical documentation. This introduces the possibility of recall and documentation bias, which may lead to the misclassification or under-reporting of subtle symptoms. Finally, the lack of longitudinal follow-up limits our ability to assess disease progression and the stability of psychiatric findings over time.

Future research should prioritize prospective, multi-centre designs with standardized data collection, broader population sampling to reduce selection bias, and repeated assessments to explore how genetic, environmental, and healthcare-related factors interact across the full trajectory of HD.

## 5. Conclusions

This article presents data from our single centre over a 20-year period. Our results indicate a diagnostic delay between symptom onset and genetic confirmation, despite most patients having a positive family history. This delay may be attributed to the increased availability of genetic testing in our country over the past decade. Additionally, the higher prevalence of psychiatric symptoms among female patients highlights the need for a personalized, gender-specific approach. Enhancing awareness among healthcare professionals and expanding access to genetic counselling could support earlier diagnosis and intervention, ultimately improving patient outcomes and quality of life.

## Data Availability

The original contributions presented in this study are included in the article/Supplementary Materials. Further inquiries can be directed to the corresponding author.

## Supplementary Materials

The following supporting information can be downloaded at: https://www.mdpi.com/article/10.3390/neurolint17060095/s1, Table S1: Data of the included patients.

## Abbreviations

The following abbreviations are used in this manuscript:

HD	Huntington’s Disease
HTT	Huntingtin gene
CAG	Cytosine-Adenine-Guanine (trinucleotide repeat)
UHDRS	Unified Huntington’s Disease Rating Scale
TMS	Total Motor Score
TFC	Total Functional Capacity
DCL	Diagnostic Confidence Level
MMSE	Mini-Mental State Examination
BDI-II	Beck Depression Inventory, Second Edition
PCR	Polymerase Chain Reaction
(18)F-FDG PET	Fluorine-18 Fluorodeoxyglucose Positron Emission Tomography
MRI	Magnetic Resonance Imaging
SPSS	Statistical Package for the Social Sciences
CI	Confidence Interval
ALS	Amyotrophic Lateral Sclerosis
